# Evolutionary scaling of maximum growth rate with organism size

**DOI:** 10.1038/s41598-022-23626-7

**Published:** 2022-12-30

**Authors:** Michael Lynch, Bogi Trickovic, Christopher P. Kempes

**Affiliations:** 1 Biodesign Center for Mechanisms of Evolution, Arizona State University, Tempe, AZ 85287 USA; 2 The Santa Fe Institute, 1399 Hyde Park Road, Santa Fe, NM 87501 USA

**Keywords:** Evolutionary theory, Cell growth

## Abstract

Data from nearly 1000 species reveal the upper bound to rates of biomass production achievable by natural selection across the Tree of Life. For heterotrophs, maximum growth rates scale positively with organism size in bacteria but negatively in eukaryotes, whereas for phototrophs, the scaling is negligible for cyanobacteria and weakly negative for eukaryotes. These results have significant implications for understanding the bioenergetic consequences of the transition from prokaryotes to eukaryotes, and of the expansion of some groups of the latter into multicellularity. The magnitudes of the scaling coefficients for eukaryotes are significantly lower than expected under any proposed physical-constraint model. Supported by genomic, bioenergetic, and population-genetic data and theory, an alternative hypothesis for the observed negative scaling in eukaryotes postulates that growth-diminishing mutations with small effects passively accumulate with increasing organism size as a consequence of associated increases in the power of random genetic drift. In contrast, conditional on the structural and functional features of ribosomes, natural selection has been able to promote bacteria with the fastest possible growth rates, implying minimal conflicts with both bioenergetic constraints and random genetic drift. If this extension of the drift-barrier hypothesis is correct, the interpretations of comparative studies of biological traits that have traditionally ignored differences in population-genetic environments will require revisiting.

Generalizations about phenotypic evolution that transcend the Tree of Life are rare, in part because few traits are exhibited across all lineages, and for those that are, the necessary comparative and empirical data are often scant. Most notable are the scalings of mass-specific metabolic rate and maximum population-growth rate with body mass. Log–log plots of such data, often referred to as power-law functions, generally appear linear with slopes commonly in the range of minus one-third to minus one-quarter^1–6^.

These statistical relationships have motivated numerous explanatory hypotheses invoking adaptive tradeoffs or structural constraints such as surface area : volume and fractal delivery-system limitations^5,7,8^. Although it has been acknowledged that metabolic-scaling relationships vary among major taxa (e.g., bacteria vs. unicellular eukaryotes vs. metazoans), the proposed explanations still rely on group-specific constraint arguments^9–12^ or ignore them entirely^13,14^. Only in a few cases^15^ have the presumed constraints been evaluated experimentally, and observations in conflict with the proposed hypotheses remain^16^.

Biophysical/bioenergetic constraints and tradeoffs need not be the only determinants of phylogenetic scaling relationships. A second general pattern across the Tree of Life is the inverse scaling of the mutation rate with the effective population size ($N_e$) of a species^17^. The mechanism driving this pattern is thought to relate to the reduced efficiency of selection for molecular refinement that results from the increase in random genetic drift associated with decreased $N_e$, a specific manifestation of the drift-barrier hypothesis^18,19^. In effect, as $N_e$ declines, the increased noise associated with drift makes it progressively more difficult for natural selection to promote weak anti-mutator alleles. Hamilton’s theory of senescence^20^, which postulates reduced efficiency of selection on alleles at late-acting loci, is another invocation of a drift barrier^21^.

These contrasting kinds of explanations for broad phylogenetic patterns highlight quite different perceptions of the mechanisms limiting the power of natural selection. Traditional metabolic-scaling and bioenergetic-tradeoff arguments assume that organismal performance is strictly constrained by the physical makeup of biology, with no genetic barriers to natural selection arising prior to hitting design limits. In contrast, the drift-barrier hypothesis postulates that constraints on population sizes, underlying genetic systems, and the distribution of mutational effects dictate the level of achievable performance.

Here, we show how the maximum capacity of a species to incorporate biomass per unit time ($g_{\text{max}}$, the maximum interval-specific growth rate of individual biomass achievable across life stages within a species) scales with adult organism size ($B_a,$ in units of mass). The resultant pattern defines the apparent upper limit to the rate of biomass production achievable in nature across the Tree of Life. As the observed scaling exponents deviate significantly from the expectations of biophysical/bioenergetic-constraint models, we suggest a role associated with size-related shifts in the population-genetic environment, most notably a progressive reduction in the efficiency of natural selection operating on mutations of small effects in eukaryotes of increasing size. This is not to say that $N_e$ is the only factor influencing the growth potential of a species or that constraints or tradeoffs play no role. However, based on the known incidence of deleterious mutations of small effects and basic population-genetic principles, it is difficult to escape the conclusion that the phylogenetic distributions of maximum growth rates, and likely of other biological features, are subject to drift-barrier constraints.

## Results

### Scaling of maximum growth rate and body mass

There have been numerous prior summaries on the relationship between maximum population-growth rate ($r_{\text{max}}$) and organismal size^3,6,9,22^. The focus here, however, is on the maximum growth rate of individual biomass achievable at any life stage within a species. These two rates need not be equivalent, as the former is a time-averaged function of age-specific growth rates, which can in some cases be quite variable. Within multicellular species, the youngest individuals almost always exhibit the highest rates of biomass incorporation, and this $g_{\text{max}}$ is not generally equivalent to the growth rate to maturity. For unicellular species, $r_{\text{max}}$ and $g_{\text{max}}$ should be nearly equivalent, given that cell growth is typically exponential^23–28^, and will be treated as such below, although slight differences may exist if cell growth varies during the cell cycle^10^.

Mass-specific growth-rates were estimated as $[\ln (B_t{/}B_0)]{/}t$, where $B_0$ and $B_t$ are individual masses at the start and end of the time interval *t*, with the maximum value observed over different life stages (in multicellular species) retained as $g_{\text{max}}$. Such estimates were obtained from the literature for a wide range of phylogenetic groups for which growth data were available for multiple species, usually in controlled environments providing near optimal growth conditions (Supplementary Tables 1, 2, 3, 4). To reduce the noise associated with differences among studies, an attempt was made to standardize all data to a temperature of 20 °C (Supplementary Text), chosen because a large fraction of the data in some groups was gathered specifically at or very close to 20 °C. There are caveats with respect to such data, e.g., optimal growth conditions may not have been obtained in particular studies, and growth rates and body masses are estimated with some error. To reduce the sampling bias associated with the identification of upper limits, a rarefaction approach was used to account for variation in numbers of estimates within species (Supplementary Text). There still remains an approximately order-of-magnitude range of variation in $g_{\text{max}}$ estimates for any particular adult mass across phylogenetic lineages, but such sampling variation is not enough to overwhelm the patterns observed over the nearly 20 order-of-magnitude range of variation of adult masses in this study, which involves 934 taxa distributed over 18 phylogenetic groups.

**Figure 1:**
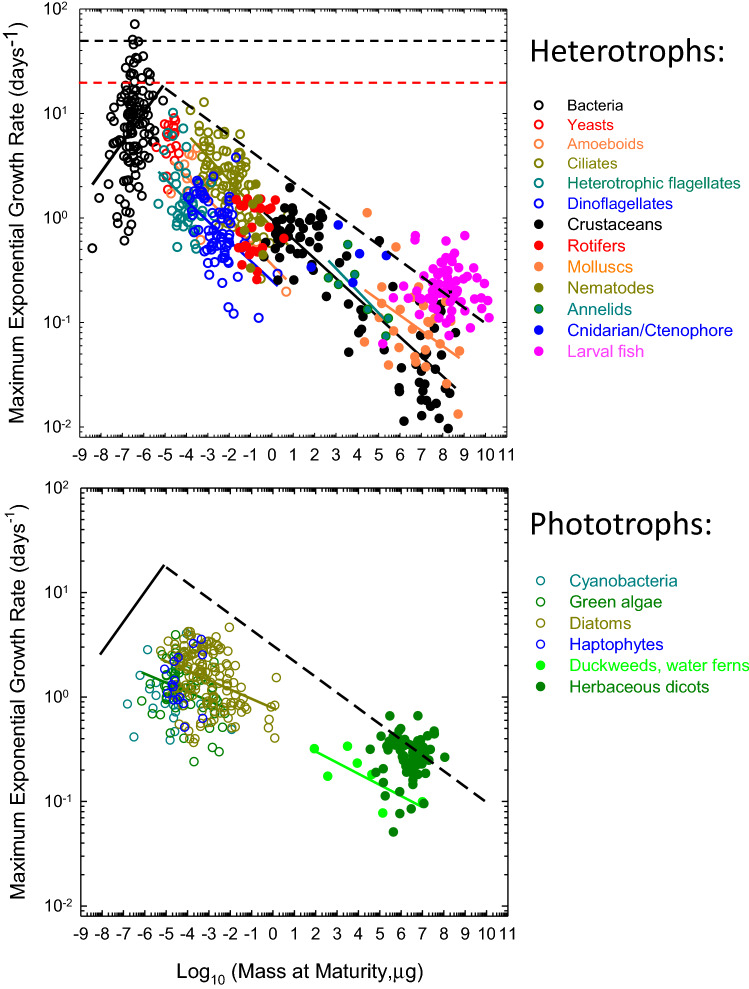
Relationship between estimates of maximum interval-specific growth rates and adult body mass for various taxonomic groups (normalized to 20 °C). Open points denote unicellular species; solid points multicellular species. The solid lines denote regressions (only shown for phylogenetic groups where significant), whereas the diagonal dashed line is the approximate upper bound to heterotrophic growth rates across all groups (as described in the text, and excluding larval fish). In the lower panel for autotrophs, the black lines from the profile for heterotrophs are added as reference points. The horizontal dashed black and red lines are the hypothetical upper bounds on growth rates dictated by the translational constraints imposed by the properties of ribosomes (as described in the “Discussion”).

Several conclusions can be drawn from the data (Fig. 1). First, as suggested previously with fewer data^9,10,29^, there is a significant positive scaling between $g_{\text{max}}$ and adult mass ($B_a$) in heterotrophic bacteria (Table 1). The maximum growth capacities of the largest bacteria are nearly an order of magnitude greater than any observations in eukaryotes of similar size. Such a pattern is inconsistent with arguments that the growth rates of large prokaryotes are compromised by the relative lack of surface area for membrane bioenergetics^30^. The repeated finding that the growth of biomass of individual bacterial cells follows an exponential trajectory (references above), and several other observations^9,10,29,31^ are also contrary to expectations for bacterial cells confronted with surface-area limitation.

**Table 1:** Estimates of the intercepts and slopes of least-squares regressions involving $\log _{10}$ transformations of $g_{\text{max}}$ and adult dry mass, in units of days${}^{-1}$ and μg, respectively.

Group	Intercept	Slope	$r^2$	*P*	*n*
**Unicellular heterotrophs**					
Bacteria	2.722 (0.469)	0.286 (0.072)	0.121	0.0001	118
Amoebozoa	− 0.446 (0.140)	− 0.210 (0.041)	0.634	0.0001	17
Ciliates	− 0.088 (0.063)	− 0.225 (0.029)	0.406	< 0.0001	90
Dinoflagellates	− 0.600 (0.111)	− 0.190 (0.043)	0.225	< 0.0001	90
Heterotrophic flagellates	− 0.645 (0.323)	− 0.205 (0.079)	0.141	0.0130	44
Yeasts	1.344 (0.853)	0.131 (0.180)	0.032	0.4762	18
**Multicellular heterotrophs**					
Annelids	0.160 (0.326)	− 0.214 (0.078)	0.601	0.0406	7
Crustaceans	− 0.013 (0.058)	− 0.188 (0.012)	0.733	< 0.0001	86
Molluscs	− 0.053 (0.363)	− 0.146 (0.053)	0.226	0.0106	28
Nematodes	0.008 (0.286)	0.093 (0.360)	0.009	0.8042	9
Rotifers	− 0.058 (0.081)	0.064 (0.080)	0.023	0.4344	29
Larval fish	− 0.710 (0.219)	0.006 (0.027)	0.001	0.8566	68
**Phototrophs**					
Cyanobacteria	− 0.113 (0.259)	− 0.025 (0.052)	0.010	0.6378	25
Chlorophytes	− 0.297 (0.137)	− 0.087 (0.032)	0.104	0.0092	64
Diatoms	− 0.107 (0.062)	− 0.090 (0.019)	0.130	< 0.0001	150
Haptophytes	0.492 (0.487)	0.082 (0.113)	0.036	0.4781	16
Duckweeds	− 0.308 (0.186)	− 0.107 (0.042)	0.558	0.0538	7
Herbaceous dicots	− 0.722 (0.230)	0.022 (0.036)	0.006	0.5451	68

Standard errors are in parentheses. Results are given only for phylogenetic groups where the number of species with available data exceeds five. The heterotrophic flagellate grouping is exclusive of dinoflagellates.

Second, although negative scaling of growth rates with organism size has been suggested previously for several groups of unicellular eukaryotes^9,10,29,32^, this can now be seen to be more broadly generalizable. Contrary to the situation in prokaryotes, in all cases, the regression is either significantly negative or, in cases where sample sizes are small or the size range is narrow, not significantly different from zero (Table 1). For the five unicellular eukaryotic groups of heterotrophs, the mean regression coefficient (optimally weighted with the inverse of the sampling variances of the individual coefficients) is $-0.208$ (SE = 0.020), whereas that for the invertebrate groups is $-0.181$ (SE = 0.011). Thus, the data for this broad set of heterotrophs are consistent with the view that $g_{\text{max}}$ declines approximately as the $-0.2$ power of adult mass. There is no tendency for the regression slope to change with organism size, with a nearly three order-of-magnitude overlap in the range of masses of unicellular and multicellular organisms. Larval fish have not been included in these analyses, as they are generally provisioned with preprocessed food in the yolk sac, but they still exhibit a nearly 100-fold reduction in maximum growth potential relative to what is achievable in unicellular eukaryotes (Fig. 1).

Third, although the size-scaling relationships are consistent across heterotrophic groups, there are significant differences in the elevations of the curves. In particular, $g_{\text{max}}$ for flagellates and amoeboid forms are several fold lower than those for ciliates, crustaceans, rotifers, and molluscs (Fig. 1). The reasons for these baseline differences are unclear, but do not seem to reflect major dietary differences, as all are fairly indiscriminate particle feeders. One caveat in interpreting such elevational differences concerns the nature of temperature correction of the data, which was applied to $g_{\text{max}}$ but not to $B_a$, as is typical in almost all such studies. Although unicellular species appear to increase in size with decreasing temperature, as much as 25% with a 10 °C decline in temperature^33–35^, the data are sparse, and there is uncertainty on the matter in multicellular species^36^. However, provided there is not a strong systematic difference in the response of $B_a$ to temperature, the lack of correction for such effects might influence the intercepts, but the slopes of these regressions are expected to be robust to such effects.

Fourth, taking all of the data together, the approximate upper bound to the growth rates of the 423 heterotrophic eukaryotes in the analysis is $g^*_{\text{max}}\simeq 3.1B_a^{-0.15}$ (in units of days$\ {}{-1}$, scaled to 20 °C), where adult body mass $B_a$ is in units of μg dry weight. This function is based purely on visual inspection, as it is unclear how to precisely estimate a general upper bound to sets of data that themselves are upper-bound estimates, but excluding the data for larval fishes, it does envelope $\sim 99\%$ of the data. The overall implication is that a 100-fold increase in $B_a$ is accompanied by $\sim 30\%$ reduction in the maximum achievable growth rate.

Fifth, contrary to the situation for heterotrophs, phototrophs exhibit a general negative scaling relationship between $g_{\text{max}}$ and $B_a$ across the bacterial and eukaryotic domains. Using a much smaller set of taxa than applied here, it has been suggested that there is a shift in the growth-rate/cell-size scaling in phytoplankton (like that noted above for heterotrophic bacteria and eukaryotes)^37–39^, but we find no statistical support for this argument. For photosynthetic bacteria (cyanobacteria), the scaling is not positive, and the $g_{\text{max}}$ estimates for individual species are on average nearly an order of magnitude below those for heterotrophic bacteria. The overall weighted regression coefficient for phototroph groups is $-0.068$ (SE = 0.013), and declines to $-0.084$ (0.014) if herbaceous plants (for which estimates of $B_a$ are least reliable) are excluded from the analysis, less than half that observed for heterotrophic eukaryotes. The net effect of this shift in scaling is that some large phototrophs have $g_{\text{max}}$ in excess of the heterotrophic eukaryotic upper bound, although as in larval fish, this may be a consequence of maternal provisioning in the youngest, seedling stages.

### The challenges of constraint/tradeoff hypotheses

Nearly all prior attempts to understand organismal scaling features have focused on arguments invoking biophysical constraints or evolutionary trade-offs, neither of which explain the eukaryotic data presented above. Focusing first on biophysical constraints, two competing classes of hypotheses postulate scaling relationships that are too steep to be reconciled with the patterns in Fig. 1. One view is that, with increasing size, the growth of unicellular organisms becomes progressively limited by nutrient uptake due to unfavorable surface:volume ratios^40^. If biosynthetic demands scale with volume, and nutrient-uptake capacity scales with surface area, mass-specific growth rate should scale with the $-1{/}3$ power of cell volume (but see below).

An alternative set of biophysical models suggests $-1{/}4$ power-law scaling, although not always for the same reasons. The most prominent of these focuses on the presumed consequences of organismal fractal delivery systems in multicellular organisms^5,7^. Questions have arisen regarding the mathematical assumptions underlying the models^41–45^, and their relevance to unicellular organisms remains unclear. Nonetheless, an alternative nutrient-delivery model assuming transport from a single point source still leads to $-1{/}4$ scaling^8,46,47^. Notably, however, drawing from comparative data in protists, Fenchel^11^ found a power-law relationship between cell surface area and volume with an exponent close to 3/4, as shapes of cells with increasing volumes shift to flatter forms; this alters the expected $-1{/}3$ scaling under the surface-area constraint model to $-1{/}4$, leaving the scaling predictions of these two models ambiguous.

The key issue here is that all of these biophysical models predict scaling exponents in the range of $-1{/}4$ to $-1{/}3$, which is much stronger than the $-1{/}10$ (autotrophs) to $-1{/}5$ (heterotrophs) power-law scaling observed for $g_{\text{max}}$. Might there be some systematic statistical bias causing the observed regression coefficients for $g_{\text{max}}$ to be less extreme than the expectations under biophysical models? Random measurement errors in dependent variables (in this case $g_{\text{max}}$) cause a reduction in accuracy but do not induce bias in regression estimates. However, sampling variance of the independent variable (organism size) can lead to estimated regressions somewhat flatter than true parametric relationships. Letting $\sigma ^2 _E$ be the measurement-error variance of individual size measures, $\sigma ^2_T$ be the variance among true values, and $x = \sigma ^2 _E {/}\sigma ^2_T,$ it can be shown that an expected ratio of true to observed regression coefficients, $\beta _T{/}\beta _{obs}$, requires the ratio of the standard errors of measures to be $\sqrt{x} = (\beta _T{/}\beta _{obs}) - 1$. Considering the least extreme biophysical-constraint case, if $\beta _T = -0.25,$ $\sqrt{x} = 0.45$ is required to alter the expected slope to $\beta _{obs} = -0.20$, and $\sqrt{x} = 1.22$ to alter it to $\beta _{obs} = -0.10$. Thus, given the many orders of magnitude over which the size data range, it is implausible that the low values of the observed regression coefficients relative to constraint-model expectations is a consequence of errors in estimation of $B_a$.

Finally, we consider the matter of evolutionary tradeoffs between alternative life-history traits. Life-history theory^48,49^ generally postulates that an enhanced performance of one trait comes at the expense of others, e.g., tradeoffs involving investment in growth versus reproduction, and offspring size versus number. Implicit in tradeoff hypotheses is a zero-sum game assumption, such that different organisms have the same fixed amount of resources, *C*, which are partitioned (for two traits) as either $C = A + B$ or $A \cdot B,$ so that any increase in *A* must be compensated by a decrease in *B*.

It is unclear how tradeoff theory would apply to the maximum interval-specific growth rate. Our focus is on the total rate of assimilation into biomass, not on how it is partitioned into alternative uses (e.g., digestive vs. defensive structures), with $g_{\text{max}}$ being analogous to *C* and largely orthologous to the tradeoffs that life-history theory focuses on. For unicellular species, there is no conflict between growth and reproduction, nor between offspring size and number. Each individual simply doubles in size per generation, and then produces two offspring equivalent in size (with the exception of budding yeasts). Thus, in bacteria, larger-celled species grow and reproduce faster.

for unicellular eukaryotes, there is a decline of maximum growth rate with increasing cell size, but we are unable to contrive a tradeoff model that would explain why, over a wide range of phylogenetic groups, large-celled species should be consistently selected to assimilate total biomass more slowly than smaller ones; under conventional tradeoff theory, growth reduction would be compensated by increased investment in alternative functions, but there is no obvious gradient of this sort in the organisms in this study. Lipids are energetically costly, but the relative investment in plasma membrane declines with increasing size, owing to the reduction in the surface area:volume ratio. For multicellular eukaryotes, there can be a separation between investment in growth and reproduction, but in no case does reproductive effort detract from the $g_{\text{max}}$ estimates reported here, as the measures are generally from life stages prior to an investment in reproduction. Our demonstration that the maximum rate of biomass assimilation varies among organisms is agnostic with respect to how energy is partitioned, and is neither in conflict with life-history tradeoff theory nor can be explained by it.

This being said, a different sort of physical-constraint argument may be relevant to the pattern in heterotrophic bacteria, e.g., increasing fractional volumetric requirements of nonscalable components such ribosomes, cell walls, membranes, and the nucleoid in very tiny cells^10,37^. Under this view, bacterial $g_{\text{max}}$ is assumed to be close to the maximum level of achievable perfection conditional upon these structural limitations, although the expected magnitude of growth-rate scaling has not been generated from first principles (but see Ref^50^), and the pattern is not observed in phototrophs.

### Evolutionary-genetic considerations

These shortcomings of constraint and tradeoff models in explaining the data motivate the consideration of alternative models. As noted above, an implicit assumption underlying all prior scaling hypotheses is that the ability of natural selection to refine complex morphological and life-history features is effectively unlimited, up to the constraints of biophysics/bioenergetics. Here, we suggest that a population-genetic constraint—a gradient in the efficiency of selection across the Tree of Life—contributes to the patterns observed in Fig. 1.

One of the central tenets of evolutionary genetics is that selection cannot eradicate a deleterious genomic variant unless its disadvantage exceeds the power of genetic drift (which equals the inverse of the effective population size, $1{/}N_e$ in a haploid population, and half that in a diploid)^51^, which we call the drift barrier. Thus, to define the reach of selection in different phylogenetic contexts, we need to know how $N_e$ varies among lineages and whether significant fractions of deleterious mutations have small enough effects to be vulnerable to passive fixation by random genetic drift in some lineages but not in others.

Under the assumption of positive selection for growth rate, for the drift-barrier to contribute to the decline in $g_{\text{max}}$ with increasing adult body mass in eukaryotes, $N_e$ must be negatively associated with $B_a$. The long-term average $N_e$ for a species can be estimated by noting that under drift-mutation equilibrium, average nucleotide heterozygosity at silent sites (in protein-coding genes) provides an estimate of $2N_eu$ in haploid species (and $4N_eu$ in diploids), with *u* being the base-substitution mutation rate per nucleotide site per generation^52^. Using direct estimates of *u* available for dozens of species^17,53^, estimates of $N_e$ (in millions of individuals) disentangled from such diversity measures yield the empirical relationship

1 $$\begin{aligned} N_e \simeq 5 B_a ^{-0.2}, \end{aligned}$$

where $B_a$ is in units of μg^54^. Although weak selection on silent sites can cause bias in $N_e$ estimates by factors of up to three-fold in either direction^53^, this is not sufficient to obscure the approximately four order-of-magnitude decline in average $N_e$ over a 17 order-of-magnitude range in $B_a$ in eukaryotes. There are numerous reasons why $N_e$ only weakly reflects absolute population sizes (Chapter 3 in Ref^52^). Even in bacteria, $N_e$ does not exceed $10^9$, presumably because the primary determinant of the power of drift in species with high abundances is the selective interference resulting from chromosomal linkage rather than numbers of individuals.

We now turn to the strength of selection operating on de novo mutations. To explain the approximately continuous scaling observed in Fig. 1, the distribution of fitness effects must be broad enough that some fraction remains vulnerable to fixation by drift over the full range of eukaryotic $N_e$. Numerous lines of evidence are qualitatively consistent with this scenario. For example, studies of serially bottlenecked mutation-accumulation lines across diverse species consistently reveal a slow per-generation decline in growth rate and other fitness traits, in accordance with a strong predominance of mutations with deleterious effects^55,56^. Further details on the mutational distribution of fitness effects, inferred indirectly from studies on the site-frequency spectra of segregating alleles (mostly in animals), commonly suggest that 10 to 40% of mutations have deleterious effects $<10^{-5}$^57–61^. The mode of this pool of mutations with small effects is typically inferred to be near (if not at) 0.0, and there are theoretical reasons for expecting this to be the case^62^. Most of these population studies focus only on nonsynonomous sites in protein-coding sequence, and comprehensive studies of the effects of single amino-acid substitutions across the lengths of proteins yield similar conclusions^63–68^. However, the genome-wide (including noncoding DNA) distribution of fitness effects of de novo mutations is certain to be even more skewed towards zero than typically inferred. For example, analyses in diverse organisms suggest that *s* is generally on the order of $1{/}N_e$ for suboptimal nucleotides at the subset of $\sim 25\%$ synonymous sites in protein-coding genes^53^.

The fitness consequences of some kinds of mutations can be ascertained from first principles. For example, the cost of synthesizing a single-base insertion of nontranscribed DNA $\simeq 100$ ATP hydrolyses^29^. Noting that the cost of constructing an *E. coli*-sized cell $\simeq 10^{10}$ ATPs, the selective disadvantage of adding a 1-bp insertion of otherwise nonfunctional DNA is then $\simeq 10^{-8}$. Equivalent to the additional fractional time required to harvest energy for offspring cell production^54,69^, this small effect is just visible to selection in bacteria with $N_e \simeq 10^8$. On the other hand, for a 100-fold larger cell, such as yeast, the selective disadvantage of a 1-bp insertion will be more on the order of $10^{-10}$ and therefore largely immune to selection. For still 10-fold larger cells, common in multicellular species, the selective disadvantage per single-nucleotide insertion drops to $\sim 10^{-11}$. Thus, with $N_e \simeq 10^6$ (on the large end for multicellular species), an insertion must be $>10$ kb in length (costing the cell $\sim 10^6$ ATPs) to be vulnerable to purging by selection on the basis of bioenergetic cost. These observations are relevant given the $\sim 1000$-fold increase in genome size (mostly due to noncoding DNA) with increasing organism size from bacteria to multicellular eukaryotes^70^. Similar arguments can be made for amino-acid substitutions with otherwise nonfunctional consequences. With the deviation between many amino-acid pairs being a bioenergetic cost of $<10$ ATPs per residue^71,72^, the inferred selective disadvantages associated with biosynthesis will often be in the domain of effective neutrality in small-$N_e$ species.

Thus, the existence of a large pool of mutations with deleterious effects small enough to allow fixation in some lineages but large enough to ensure removal by selection in others is not in doubt. For deleterious-mutation accumulation to be ruled out as a contributor to patterns of growth-rate scaling across the Tree of Life, all mutations would have to have effects $>10^{-4}$ (the inverse of the smallest observed $N_e$), which is biologically implausible. Should the drift-barrier hypothesis be the correct explanation of the eukaryotic patterns observed in Fig. 1, three inferences can be made.

First, the fitness effects of the mutations involved must be very small. Letting $s^*= 1{/}(2N_e)$ denote the approximate value of the deleterious effects of mutations below which selection is ineffective (for diploids), from Equation (1), the critical cutoffs are $s^*\simeq 10^{-8}$ and $10^{-5}$ for the lower and upper ranges of eukaryote $B_a$ in Fig. 1. Note that we are specifically concerned with the subset of *growth-reducing* mutations that reside in this window, and that there need not be a 1:1 relationship between the growth and fitness effects of individual mutations. Indeed, one can appreciate the difficulty with the drift-barrier hypothesis if the growth-altering effects of mutations were identical to fitness effects by considering $s^*= 10^{-8}$ noted above for the smallest eukaryotic cells. From the $-0.2$ scaling relationship of $g_{\text{max}}$ with $B_a$, it follows that an order of magnitude increase in $B_a$ leads to a 37% reduction in $g_{\text{max}}$. Assuming that growth-reducing effects operate in a multiplicative manner, and letting *n* denote the number of such effects, then to achieve this level of reduction, we require approximately $(1 - 10^{-7})^n = 0.63,$ which implies $n \simeq 5 \times 10^6$, about half the total number of nucleotide sites in the genomes of the smallest unicellular eukaryotes. If, however, at this extreme end of the size distribution, there is a class of conditionally neutral mutations, $s^*\simeq 1/(2N_e),$ with growth-reducing effects $\Delta g$ on the order of $10^{-6}$ or larger, then the necessary number of factors to explain the data becomes more palatable, as fewer than 500,000 nucleotide sites would be required.

Second, for heterotrophic eukaryotes, both $g_{\text{max}}$ and $N_e$ scale with the $-0.2$ power of $B_a$, implying that for this group $g_{\text{max}}$ scales linearly with $N_e$, which is remarkably similar to the scaling of genome-replication accuracy and effective population size^17^. The shallower response of $g_{\text{max}}$ to $B_a$ for phototrophs, with coefficient $-0.1$, might suggest a scaling of the former with the square root of $N_e$, but the regression leading to Equation (1) relies almost entirely on heterotrophs, so it is premature to draw this conclusion. Nevertheless, as phototrophs are lower on the food chain, it is plausible that the lower sensitivity of $g_{\text{max}}$ to $N_e$ in this group is a consequence of a less extreme gradient of $N_e$ and $B_a$ than in heterotrophs.

Third, the continuous exponential decline of $g_{\text{max}}$ with increasing $B_a$ implies an inverse relationship between the numbers and average effects of growth-reducing mutations subject to drift. This follows from the simple fact that the fractional reduction in growth rate must be essentially constant for each incremental change in $B_a$ over the entire size range of observed data. Letting $\Delta _g$ be the critical average growth-reducing influence of an effectively neutral mutation at a particular point on the size gradient, and *n* be the the number of genomic sites subject to such mutations, then the total exposable growth load at that particular point is $1 - (1 - \Delta _g)^n \simeq n\Delta _g$. As genome size increases with $B_a$, constant $n\Delta _g$ requires that for each increment in $B_a$ both $\Delta _g$ and the incremental expansion of growth-influencing genomic sites (*n*) remain constant or that $n \propto 1/\Delta _g$, i.e., as the size-related increment in genomic sites increases, the average associated growth-influencing effect per site declines.

Note that the distribution of effects referred to here is not strictly the same as the distribution that would be observed in any particular species, although this possibility is not ruled out. All that is required is that as $B_a$ increases (and $N_e$ decreases), a pool of growth-reducing mutations with constant net effects emerge per unit increment in $B_a$. This could, in part, be a consequence of the emergence of genomic sites with very small effects that do not even exist in small-$B_a$ species (for reasons noted above). As the groups of organisms in Fig. 1 are substantially different physiologically and morphologically, the nature of mutations contributing to such behavior could be quite different among phylogenetic lineages.

### The evolutionary distribution of a trait under persistent directional selection

We now turn to a more formal analysis of the previous points. The lack of precise knowledge of the joint distribution of mutational effects on growth rate and fitness, and the lack of an explicit mapping between $g_{\text{max}}$ and fitness, compromise our ability to make more than a qualitative statement about the expected size-scaling of growth rate under the drift-barrier hypothesis. What can be shown, however, is the plausibility of a substantial and near continuous decline of growth rate over the full range of adult sizes.

To clarify the reasoning underlying this assertion, we performed computer simulations in a haploid Wright–Fisher framework for the situation in which there are one or two classes of linked biallelic genomic sites, one with Major (*M*) and the other with minor (*m*) effects on fitness, numbering $L_M$ and $L_m$, respectively, with the deleterious effects of mutations denoted as $s_M$ and $s_m$ (Supplemental Text). Although there must be an essentially continuum of genomic-site effects in most organisms, a focus on the two-state case allows a more transparent presentation of the key issues.

There are numerous ways in which individual mutations might influence total fitness, but for illustrative purposes, we assume a multiplicative fitness function in which mutations act independently,

2 $$\begin{aligned} W = e^{-s_M (L_M - L_M^+) - s_m (L_m - L_m^+)}, \end{aligned}$$

where $L_M^+$ and $L_m^+$ denote the numbers of genomic sites harboring beneficial alleles (assuming haploidy), such that $W=1$ when all sites are occupied by beneficial alleles. As a means of summarizing the weighted density of beneficial alleles in an individual, we then define a measure of performance on a scale of 0 to 1,

3 $$\begin{aligned} z = \dfrac{ L_M^+ s_M + L_m^+ s_m }{ L_M s_M + L_m s_m }, \end{aligned}$$

and evaluate how the population mean, ${\overline{z}},$ evolves with changes in $N_e$. (Under a single-effect model, performance is simply equal to the fraction of sites containing a $+$ allele). We emphasize that the index of mean performance with respect to fitness need not be equivalent to growth rate, nor a linear function of it, although we expect the two to scale monotonically.

The limits of ${\overline{z}}$ at very small and very high $N_e$ are clear. If the effective population size is sufficiently small that $s_M \ll 1/N_e$, selection will be overwhelmed by the vagaries of genetic drift, and all sites will have expected $+$ allele frequencies of $u_{01} / (u_{10} + u_{01}),$ where $u_{10}$ and $u_{01}$ are respectively the rates of deleterious and beneficial mutation. This neutral frequency is equivalent to the minimum performance at low $N_e$. In contrast, for the opposite extreme in which $s_m \gg (1/N_e)$, the expected frequencies of deleterious alleles are approximately $u_{10} / (u_{10} + u_{01} + s)$, where $s = s_M$ or $s_m$ for the two classes, which is near 0.0 provided the strength of selection substantially exceeds that of mutation.

The central issue is then the degree to which specific classes of deleterious alleles move from the domain of accumulation by mutation pressure alone to the domain of effective purging by selection as $N_e$ increases. The problem is solvable analytically for the special case of free recombination, using the general expression of Kimura et al.^73^, but although progress has been made^19,74^, an analytical solution for linked alleles is not available, and reliable results require computer simulations.

**Figure 2:**
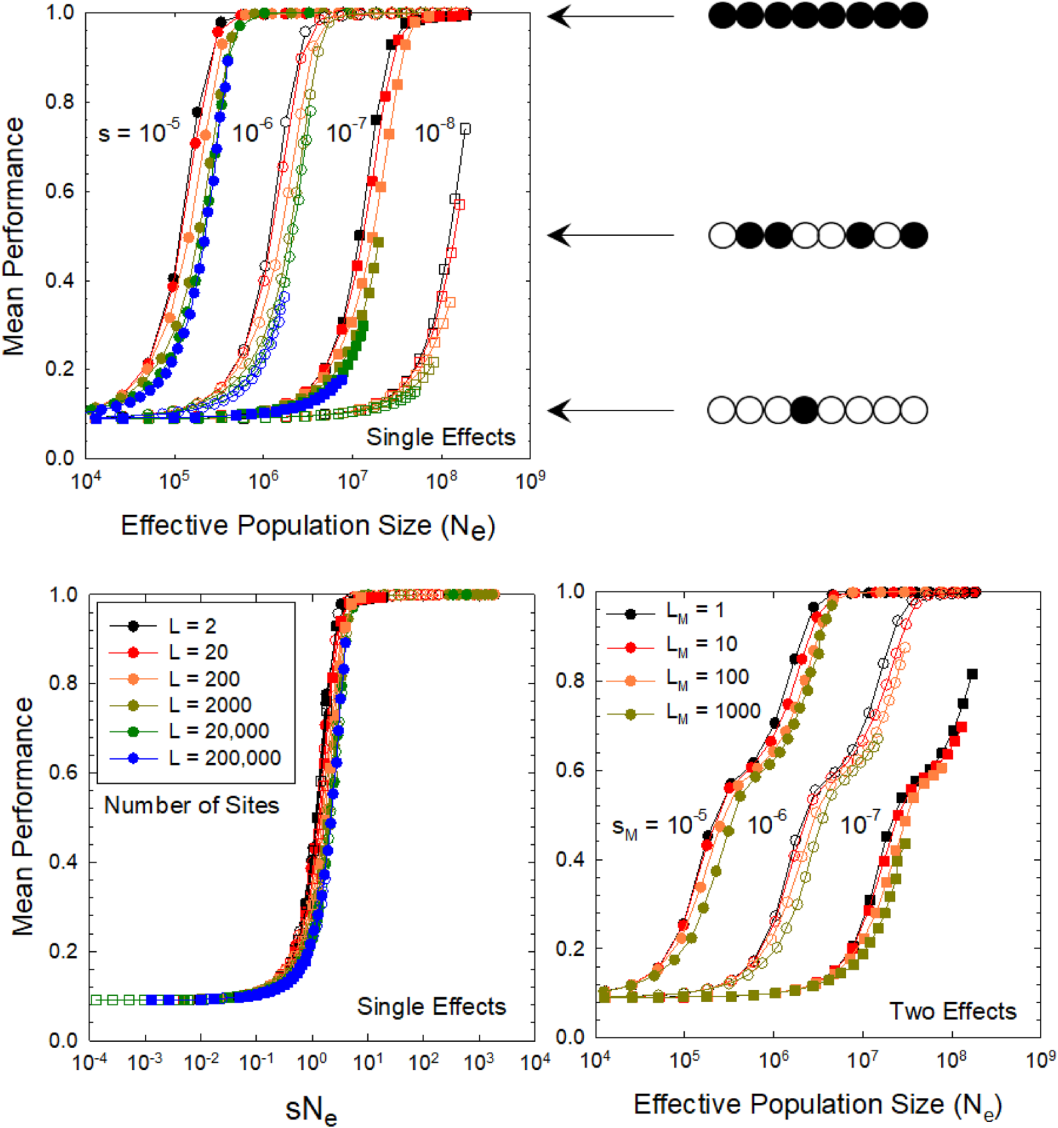
Mean performance (on a 0 to 1 scale) as a function of the effective population size ($N_e$), the selection coefficient (*s*), and the size of linkage groups, for the case of a biallelic model with the mutation rate to the beneficial allele being $10\times$ the reciprocal rate. All results denote the selection-drift-mutation equilibrium performance (as described in the main text, but equal to the mean frequency of $+$ alleles for the case of single effects). (Upper left) Results are given for the situation in which all sites have equal mutational effects, for four values of the selection coefficient *s* (bundles of curves) and six linkage-block sizes [color coded as in the inset of the lower left panel]. The cartoons to the right denote arbitrary stretches of linked sites with different fractions of sites containing $+$ alleles (solid balls), increasing as mean performance increases. As *s* declines by a factor *x*, the curves shift to the right in an essentially constant pattern, such that a specific level of performance requires an *x*-fold increase in $N_e$. (Lower left) The latter point is made by plotting the points in the upper left panel against the product $N_e s$, which leads to nearly perfectly overlapping curves. (Lower right) Results for the situation in which two types of linked sites are simultaneously selected upon: the numbers and selection coefficients associated with large-effect sites are given within the graph, whereas the small-effect sites are $10\times$ more abundant but have $10\times$ smaller selective effects, such the full performance of the system is equally distributed over the linked sets of large- and small-effect sites.

To clarify the relative roles of *s* and $N_e$ in the behavior of this system, we consider first the ideal situation in which all genomic sites have equivalent effects (Fig. 2A,B). Here, it can be seen that for any value of *s* there is a sharp inflection in mean performance as the effective population size exceeds 1/*s*, with just a slight upward shift in this pivot point with linkage groups containing larger numbers of sites. Thus, as population sizes decline, sites with increasingly large *s* become progressively enriched with deleterious alleles.

Now consider the situation in which the mutational vulnerability per linkage block is the same as in Fig. 2A, but spread equally over sites with two different effects, with the minor-effect sites being ten-fold more abundant but having ten-fold smaller *s* (consistent with the exponential distribution of fitness effects noted above). In this case, the gradient of mean performance extends over two orders of magnitude of $N_e$ (Fig. 2C), again with just a slight shift in the gradient to higher $N_e$ with linkage groups containing more sites. This altered response to $N_e$ occurs as major-effect sites first become fixed for beneficial alleles at an $N_e$ that is too small for efficient selection on the minor-effect sites. As the major-effect sites become saturated with $+$ alleles at large $N_e$, the gradient in performance continues as the minor-effect sites begin to respond to selection. Bringing in a third site type with a further ten-fold increase in abundance but a ten-fold decline in effects would extend the gradient over still another order of magnitude of $N_e$. This kind of behavior naturally arises as each decline of $N_e$ opens a new window of sites with effects $s \simeq 1/N_e$ vulnerable to fixation by deleterious alleles.

Note that the effective population sizes in Fig. 2 are not the absolute population sizes (*N*), but the inferred $N_e$ from the behavior of neutral loci linked to the selective sites in the computer simulations, as this represents the empirical $N_e$ estimates derived from silent-site heterozygosities in natural populations (noted above). When recombination rates are high (small linkage-block length, *L*), selection can operate efficiently on individual sites, but as linkage blocks become larger, selective interference among joint polymorphisms at multiple linked sites becomes increasingly important, reducing $N_e$ relative to *N*.

These linkage effects are relevant because the mean number of crossovers per chromosome arm is $\simeq 1.0$ per meiotic event in all eukaryotes^75^, whereas mean chromosome lengths increase $>100$-fold from the smallest to the largest eukaryotes^70^. The net consequence of these genetic features is an expected increase in the size of linkage blocks by 3 to 4 orders of magnitude with increasing size of eukaryotic organisms. Combined with the increased incidence of mutations with very small *s* (based on the bioenergetic considerations noted above), these linkage effects further magnify the vulnerability of large-$B_a$ species to the accumulation of mildly deleterious mutations.

## Discussion

With a goal of determining the maximum rate at which biomass can be produced across a wide swath of biology, we focus on a trait not previously considered in studies of the size-scaling of features associated with energetic performance—the maximum interval-specific growth rate. Contrary to a previous suggestion^30^, eukaryotes do not experience an energetic advantage relative to prokaryotes^29,31,76^. The most productive heterotrophic bacteria tend to be the largest ones, and these convert resources into biomass at nearly $10\times$ the rate observed in any eukaryote. Moreover, $g_{\text{max}}$ declines by $\sim 100\times$ from the smallest to the largest eukaryotic heterotrophs, and the decline is consistent both within and among groups of unicellular and multicellular species.

How far are the growth rates in Fig. 1 from the upper limit based on the foundational features of biology? An absolute upper bound on the limit to cellular growth rates can be obtained by assuming biomass to consist entirely of ribosomes and simply considering the time required for ribosomal-protein replacement. The full set of proteins per bacterial ribosome comprise $\sim 7500$ amino acids^77–79^, and generously assuming that an extended ribosome (including accessory and assembly proteins) expands this to 15, 000 amino acids, the time required for the replacement of an extended ribosome can be obtained by dividing by the translation rate. Estimates of the latter range from 3 to 20 amino acids/second^80–85^, averaging close to 12 for both prokaryotes and eukaryotes, yielding a doubling time $\simeq 0.014$ days or an upper limit to the exponential growth rate of 50/day. Eukaryotic ribosomes are $\sim 1.5\times$ larger than those in bacteria, and the $>100$ additional proteins allocated to ribosomal assembly in eukaryotes relative to prokaryotes^86^ further inflates the investment by at least another $1.5\times$, so for eukaryotes, the upper limit to growth rate is $\sim 2.25\times$ lower than that for bacteria, i.e., 20/day.

These are crude absolute upper limits, as they ignore the other structural investments that cells must make to produce offspring. Remarkably, however, although the smallest eukaryotic cells grow at rates that are $\sim 50\%$ less than the predicted eukaryotic speed limit, the fastest growing bacteria (with large cell volumes) have growth rates that extend to the upper speed limit^76^. This implies that natural selection has been capable of advancing large prokaryotic cells with the maximum possible rate of biomass production. As this upper bound is dictated by the basic properties of translational biology, regardless of the amount of energy intake, the only way to further elevate the maximum growth rate would be to reduce the size of ribosomes or increase the rate of translation. Any further increase in bacterial cell size might be advantageous in particular ecological contexts, but this would not be expected to further enhance maximum growth rates, regardless of the amount of ATP production. Smaller bacteria, with higher surface area:volume ratios and hence the potential for higher membrane-bioenergetic capacity, have reduced growth rates owing to the increased relative investment in the cell membrane itself^50^. Notably, strong selection for increased growth rate can yield a successful response in lab cultures of bacteria, but this seems always to be accompanied by an increase in cell volume and/or decrease in surface area:volume ratio^87–89^. All of these observations are quite contrary to the hypothesis that constraints on the upper limit to bacterial cell size imposed by membrane-bioenergetic limitations were solved by the evolution of mitochondria^30^.

Based on the arguments above, bacterial species may typically have large enough effective population sizes and small enough cell volumes that natural selection is capable of perceiving even the smallest possible mutational effects. However, in light of the inability of bioenergetic constraint/tradeoff models (at least as currently constructed) to explain the scaling features of the eukaryotic data, we suggest an alternative view for eukaryote growth-rate scaling. Our hypothesis postulates that by precipitating an increase in organism size, evolution of the eukaryotic cell and the subsequent emergence of multicellularity induced effective population size declines as a by-product, which in turn progressively reduced the ability of natural selection to eliminate mutations with larger and larger growth-reducing effects. In effect, this may be viewed as a tradeoff argument of a different sort—by evolving a different cell-body plan and entering different favorable ecological domains, eukaryotes passively entered a domain of reduced efficiency of natural selection.

Although this hypothesis is based on general and well-documented population-genetic and genomic properties^70^, the lack of sufficient information to derive a specific size-scaling parameter may be viewed as an unsatisfying state of affairs. It should be noted, however, that constraint-based models proposing specific scaling relationships for metabolically related traits generally do so without directly validating the operation of the presumed constraints, and lack consensus as to whether even the correct scaling coefficients have been identified. Thus, the door remains open to alternative explanations for the phylogenetic divergence of growth rates.

Further tests of the drift-barrier hypothesis for growth-rate evolution might be pursued by taking advantage of situations in which organisms of similar size have shifted into environments leading to altered $N_e$. As one potential example, consider the situation for ciliates, which have on average nearly 10-fold higher maximum growth rates than those of dinoflagellates, other heterotrophic flagellates, and amoebozoa with the same $B_a$ (Fig. 1). Although there are only a few estimates of $N_e$ for unicellular eukaryotes, those for the ciliate genera *Paramecium* and *Tetrahymena* range from 0.9 to $2.8 \times 10^8$, the highest observed values for all eukaryotes and $\sim 10\times$ or greater than those in other unicellular eukaryotes: $1.9 \times 10^7$ for the amoebozoan *Dictyostelium discoideum;* 0.8 to $1.4 \times 10^7$ for the yeasts *S. cerevisiae* and *S. pombe;* $8.9 \times 10^6$ for the diatom *Phaeodactylum tricornutum;* and 2.0 to $4.3 \times 10^7$ for the green algae *Chlamydomonas reinhardtii* and *Ostreococcus tauri*^54^. Thus, the data are consistent with the relatively high growth rates of ciliates being a consequence of their relatively low vulnerability to the vagaries of random genetic drift. Extensions of such observations to other phylogenetic groups will be required to further test this hypothesis.

For those who remain convinced that the broad phylogenetic pattern illustrated in Fig. 1 can only be a function of physical constraints or tradeoffs, there are two additional challenges. First, there is the problem that the size-dependent pattern of decline in eukaryotic $g_{\text{max}}$ is shallower than that expected under any constraint model. Although one might adopt a pluralistic view with a $-1/4$ or $-1/3$ scaling model serving as a null physical-constraint expectation, unless the power of drift were greater in smaller organisms, which is demonstrably not the case, any additional contributions from population-genetic limitations would yield an even stronger expected negative scaling than the null biophysical model, which is also demonstrably not the case. Second, there is a need to articulate a mechanism by which phenotypes can be reliably advanced to their biophysical limits in the face of a persistent onslaught of very mildly deleterious mutations, regardless of the population-genetic environment. There are no obvious reasons for why growth rates should be immune to the universal force of random genetic drift, but if this could be shown to be true, it would constitute a major challenge for the relevance of population-genetic details in interpreting patterns of long-term divergence of characters under selection.

On the other hand, given that the drift-barrier hypothesis appears to be compatible with patterns of variation for a range of other genomic and proteomic features across the Tree of Life^17,29,70,90–95^, determining whether other cell biological traits (many of which scale with organism size^96^) are causally associated with the power of random genetic drift merits consideration. If this is the case, the tradition of interpreting all phylogenetic scaling relationships as simple consequences of biophysical/bioenergetic constraints or tradeoffs will need to be revisited and integrated with population-genetic considerations.

Finally, although the observations and theory presented above were initially derived primarily for unicellular species, it is notable that the scaling features generalize to multicellular organisms. We have not attempted to extend the analyses to vertebrates, as the refined sets of observations on the very earliest interval-specific growth rates are limited and complicated in most cases by recurrent maternal provisioning. One might be concerned that the massive improvement in net growth rates to maturity that have been achieved in selective breeding programs with birds and mammals^97–101^ are inconsistent with the drift-barrier hypothesis. However, these studies do not specifically identify $g_{\text{max}}$, are often targeted to particular tissues, and invariably produce substantial negative pleiotropic effects on fitness (not because of energy-budget tradeoffs, but owing to major physiological and/or anatomical aberrations). Thus, most of the allelic variants promoted in domestication selection may be of limited relevance to the kinds of patterns noted in Fig. 1. The response to selection for overall growth improvement at the expense of all else is not inconsistent with the drift-barrier, as the mutations exploited in such studies are largely selected against in nature. Indeed, the drift-barrier hypothesis is focused on the pool of growth-reducing mutations with minor deleterious effects, not on growth-enhancing mutations with major deleterious side effects.

## Supplementary Information

Supplementary Information 1.

Supplementary Table 1.

Supplementary Table 2.

Supplementary Table 3.

Supplementary Table 4.

## Data Availability

All of the data utilized in this paper are contained and freely available in the supplementary tables.
