# Supplementary material for: Evolutionary scaling of maximum growth rate with organism size
Source: Sci Rep. 2022 Dec 30;12:22586. doi: 10.1038/s41598-022-23626-7 (PMC9803686; doi:10.1038/s41598-022-23626-7)

æ

SUPPLEMENTAL TEXT

Data set. Estimates of organism size (mean cell size for unicellular species, and size at maturity for multicellular species) and growth rates were obtained for a wide range of phylogenetic groups by extracting information from the literature (Supplemental Tables 2-4). All data were obtained from the primary literature (as opposed to being drawn secondarily from compilations in prior reviews) to minimize the inclusion of prior recording errors.

Estimates of organism size. To place the sizes of unicellular species on a scale consistent with multicellular organisms, all cell sizes are reported in units of dry weight. For the majority of species, only cell volumes are reported in the literature. However, joint data on cell volume and cell dry weight available for 68 diverse taxa (Supplemental Table 1) are highly correlated in a uniform way across phylogenetic groups (Supplemental Figure 1), and the resultant regression was used to convert cell volume to cell dry weight for the species for which direct measures of the latter were unavailable.

For multicellular species, adult sizes are typically recorded directly in units of dry weight in the literature. In cases where the reported sizes were in units of length, these were converted to dry weights from the known length-weight regressions for the species.

For a substantial number of species, independent sizes estimates were available. To minimize the influence of outliers, these were condensed to single overall estimates by first log-transforming, and then averaging and transforming back to the original scale.

Estimates of maximum growth rates. An attempt was made to only utilize growth-rate data where it appeared that the organism was grown at a saturating nutrient/food supply. However, growth rates are also functions of temperature, which can vary widely among studies. As one of the most commonly utilized culture temperatures is 20°C, we normalized all rate values to this temperature assuming a Q10 of 2.5, which is in the middle of the range typically reported for most phylogenetic groups: $g_{20} = g_T \cdot e^{0.0916(20-T)}$, where T is the temperature (in degrees C) at which the original rate measure g_T was taken.

An alternative approach to temperature correction involves the application of an equation

for Arrhenius-rate behavior, but in earlier evaluations, we found that both approaches yielded essentially identical scaling behavior. For groups with statistically significant regression coefficients, those obtained with the Q10 vs. Arrhenius approaches are: heterotrophic bacteria (0.206 vs. 0.208); amoebozoans (-0.181 vs. -0.189); ciliates (-0.209 vs. -0.220); annelids (-0.211 vs. -0.185); crustaceans (-0.182 vs. -0.193); and molluscs (-0.140 vs. -0.138).

For unicellular species, growth rates were estimated (as noted in the text) directly from the rate of cell division. For multicellular species, however, we recorded rates of growth for the stage in which this parameter takes its maximum value, as the primary focus here is on the maximum rate of biomass production for the species.

As the number of maximum growth-rate estimates varied from species to species, usually in the range of one to five, we used a rarefaction approach to reduce the bias in estimates of upper bounds caused by finite sample sizes. For 22 species for which there were five independent estimates of maximum growth rates, we estimated the maximum observed value, and then compared that with the average maxima obtained with all possible subsets of reduce sample sizes. This led to multiplicative correction factors for the upper values found in sample sizes smaller than five to allow for normalization to a sample size of five: 1.690 for samples sizes of one, 1.298 for sample sizes of two, 1.148 for sample sizes of three, 1.061 for sample sizes of four, and 1.000 for sample sizes of five. From this, it is seen that there is little need to correct for bias with sample sizes greater than four, but in the few cases for which sample sizes exceeded five, we took the average of the two highest values to allow for possible upward measurement-error bias associated with outlier values.

Population-genetic theory. Expected responses of growth-related traits to changes in population size were obtained by computer simulations, as previously described in Lynch (2020). Briefly, a Wright-Fisher construct was applied with a haploid population of size N (ranging from $\sim 10^4$ to 10^9 individuals) experiencing discrete generations with consecutive steps of mutation, selection, and random sampling for very long time, with mean phenotypes recorded periodically until the temporal distribution had stabilized. The behaviors of linkage blocks ranging from 1 to 200,000 completely linked nucleotide sites were studied, with either one or two types of sites with distinct selection coefficients.

Throughout, a multiplicative fitness function was assumed, as described in the main text,

with two allelic types (+ and −) assumed per site. After each generational episode of mutation and selection, random genetic drift was implemented by multinomial sampling of all classes containing nonzero numbers of individuals. Mutations were reversible, so that over time for any set of population-genetic conditions, a quasi-steady-state distribution of the mean number of + alleles per haplotype block was eventually reached. The primary focus here is on the equilibrium frequency of + alleles per site.

In each simulation a linked neutral locus, with the same mutation rates as the selected loci, was also monitored to yield a long-term mean estimate of the variation at silent sites and the subsequent extraction of the effective population size N_e by factoring out the mutation rate (as described in the main text).

SUPPLEMENTARY TABLES

Supplementary Table 1. Cell volume vs. cell dry weight. Where multiple references are given, the reported value is an average.

Supplementary Tables 2, 3, and 4. Summaries of data on organism size and maximum growth rates for unicellular heterotrophs, metazoans, and phototrophs.

SUPPLEMENTARY FIGURE

Supplementary Figure 1. Relationship between dry weights and volumes of individual cells. The regression line is applied to all groups simultaneously; $\log_{10}(\mathrm{DW}) = -3.244(0.040) + 0.920(0.013)\log_{10}(\mathrm{CV})$, where cell dry weight (DW) is in nanograms, and cell volume is in $\mu\mathrm{m}^3$; standard errors of the parameters are in parentheses; $r^2 = 0.99$, $n = 68$. Data taken from various sources in the literature are recorded in Supplemental Table 1.

Supplemental Figure 1. Regression of cell dry weight on cell volume for 68 species in diverse phylogenetic groups for which data exist for both traits (Supplemental Table 1). The regression line is applied to all groups simultaneously: $\log_{10}(DW) = -3.244(0.040) + 0.920(0.013) \log_{10}(CV)$; standard errors of the parameters are in parentheses; $r^2 = 0.99$.

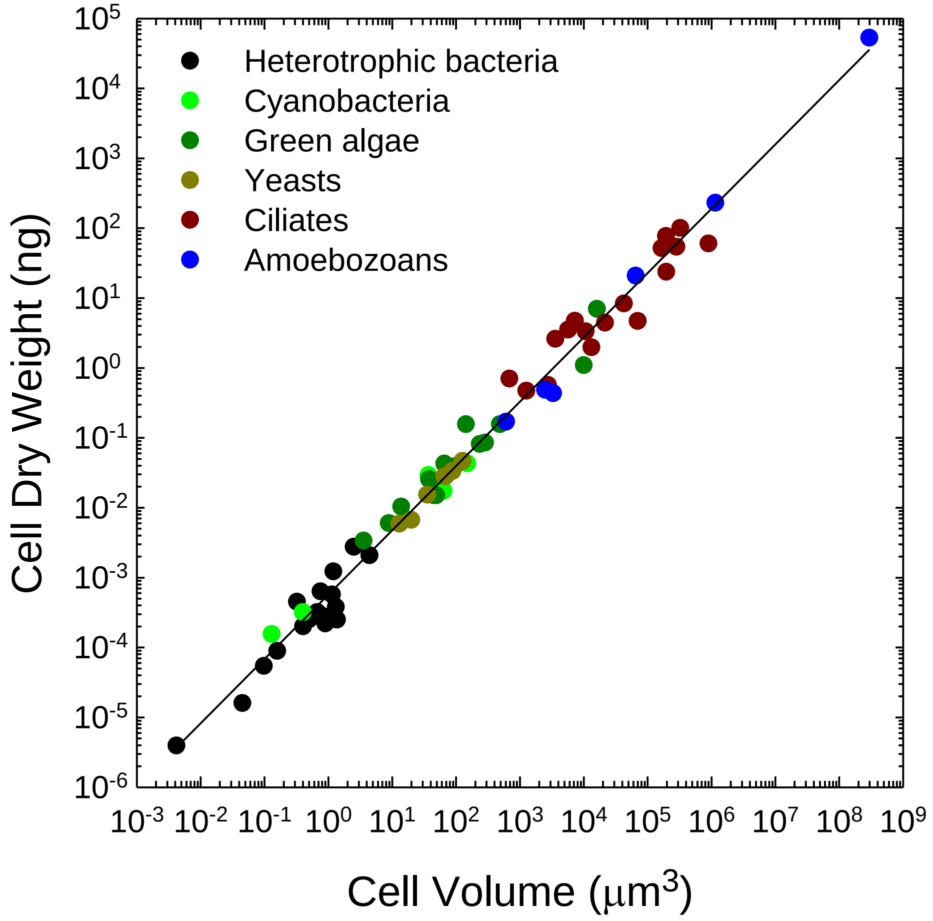

Supplement: Supplementary file 1 — Supplementary Information 1. [file 41598_2022_23626_MOESM1_ESM.pdf]
